# The use of the 1 mm laparoscope to assist in port insertion in pelvic oncological surgery

**DOI:** 10.1186/1477-7800-3-9

**Published:** 2006-04-03

**Authors:** Philippa Sangster, Thomas EJ Ind

**Affiliations:** 1 Department of Gynaecological Oncology, The Royal Marsden NHS Foundation Trust, Fulham Road, London, SW3 6JJ, UK

## Abstract

**Background:**

A 1 mm minilaparoscope (Lifeline Biotechnoligies, Florida, USA) was assessed for aiding port site insertions.

**Methods:**

Ten consecutive patients having laparoscopic procedures in a gynaecological oncology unit were included. Minilaparoscopy was feasible in all cases and was used to insert the umbilical port under direct vision in all patients. In one case, a thick band of abdominal adhesions was identified and a further lateral port site was inserted to aid their dissection.

**Results:**

The minilaparoscope correctly identified all 10 patients with peritoneal disease and identified all patients who were suitable for debulking procedures.

**Conclusion:**

Minilaparoscopy with the 1 mm endoscope appears to be safe and accurate and we feel that it has a place in helping the surgeon identify adhesions and peritoneal disease as well as assisting further port site insertion safely and with minimal complications.

## Background

Laparoscopy can be associated with a risk of visceral damage during primary trocar insertion (1). This is particularly so in oncology cases where there are likely to be adhesions and peritoneal malignancy. Microendoscopes work by transmitting light through fiberoptic bundles rather than the rigid lens-based system of traditional endoscopes. Mini laparoscopes have been used to assist port site insertion (2), we describe the use of a new 1 mm mini-laparoscope (Ovascope, Lifeline Biotechnoligies, Florida, USA) to assist primary trocar insertion during gynaecological oncology surgery.

## Methods

Ten consecutive patients who had a minilaparoscopically guided 1 mm laparoscopy are reported. Minilaparoscopy was performed using the Ovascope (Lifeline Biotechnoligies, Florida, USA). Patients included gynaecological oncology patients due to have a laparoscopy. Five patients (50%) were having laparoscopy for assessment of suitability for debulking surgery.

Following informed consent, patients were anesthetised and placed in a modified Lloyd-Davis position. Insufflation was primarily performed using a Veress needle at Palmar's point. The trocar from the 1 mm laparoscope was inserted at Palmar's point and then the mini-laparoscope was used to assess disease status and assess the presence of abdominal adhesions. Once a site for safe trocar location was identified the primary trocar was inserted.

## Results

Five patients (50%) had laparoscopy to assess suitability of debulking for ovarian cancer. One patient (10%) was having a laparoscopic colostomy formed as a treatment for bowel obstruction in recurrent metastatic endometrial cancer. One patient (10%) was having a laparoscopic ovarian cystectomy and three (30%) were having bilateral salpingo-oophrectomy. In terms of co morbidities; six of the patients had previous mid-line laparotomies, two patients had previously undergone a laparoscopy, one of the patients was obese (BMI > 30) and five patients had ovarian carcinoma.

In all the cases the mini laparoscopy was used to insert the primary port through the umbilicus under direct vision. The mini-laparoscope achieved a view successfully in 10 patients (100%). The mini-laparoscope correctly identified the sites of peritoneal disease 2 patients with omental involvement. The mini-laparoscope successfully identified the patients who were suitable for optimal debulking and showed the absence of peritoneal involvement in all five patients with ovarian cancer.

In nine patients the primary port was subsequently inserted under direct vision through the umbilicus. In one case, the mini- laparoscope identified a thick band of adhesion over the anterior abdominal wall and a further laparoscopic port was inserted laterally so that these adhesions could be dissected.

## Discussion

The ability to use the microlaparoscope to reassess patients with ovarian cancer has not been adequately investigated. One group with a small patient number (n = 8) showed that it is feasible using a 2.8 mm (5), to reduce the risk of major complications in relation to the blind insertion of the umbilical trocar as well as provide good diagnostic accuracy.

Although the technique for inserting the primary trocar in this way is not new, smaller equipment is becoming increasingly popular as is all forms of minimally invasive surgery. This is particularly so as surgeons hope to avoid larger trocar incisions and their associated complications, and the wish to perform these laparoscopic procedures in the outpatient department. Laparoscopy performed under local anesthetic in the outpatient department has also been shown to be well tolerated and safe (3). The use of laparoscopy and minilaparoscopy in both general surgery and gynaecology has been compared in terms of complications, pain and recovery (6, 7). No significant difference was seen in pain scores between groups, but the patients who underwent minilaparoscopy had shorter stays in hospital, required less analgesia and reported increased satisfaction with their wounds. However, the minilaparoscope does have limitations in standard practice. Abdominal procedures which require a high degree of manipulation, specialized instruments or larger escape hatches will require the larger, standard laparoscopic equipment.

**Figure 1:**
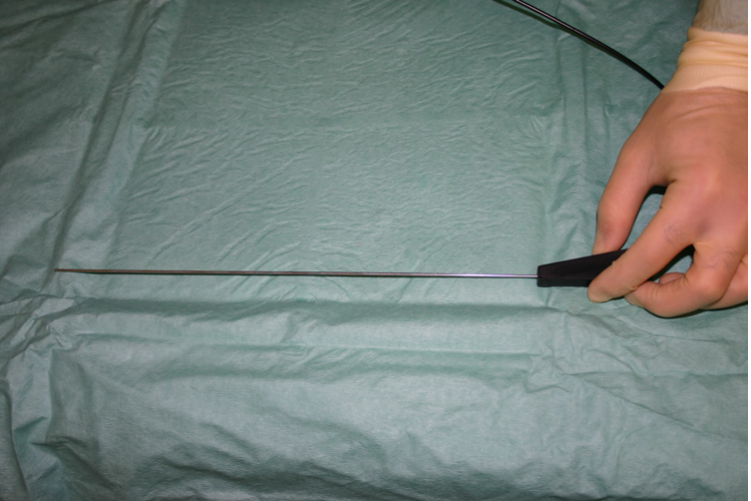
Photograph showing the 1 mm mini-laparoscope.

## Conclusion

This data demonstrates that a new 1 mm laparoscope (Ovascope, Lifeline Biotechnoligies, Florida, USA) has value in gynaecological oncology surgery. We feel that it would be a useful tool to aid primary trocar insertion and may be of value in identifying peritoneal disease prior to consideration of debulking surgery. Patients who have undergone previous surgery have a higher incidence of umbilical adhesions. High risk groups include those who have had midline or horizontal incisions for their laparotomies (4). These patients might benefit from a preliminary inspection with the microlaparoscope to rule out adhesions and therefore decrease the risk of complications.

## Competing interests

The author(s) declare that they have no competing interests.

## Authors' contributions

TEJI carried out the procedures with the minilaparoscope and conceived the study. PS collated the information and drafted the manuscript. All authors read and approved the final manuscript
